# Engineered Bone Marrow Stem Cell-Sheets Alleviate Renal Damage in a Rat Chronic Glomerulonephritis Model

**DOI:** 10.3390/ijms24043711

**Published:** 2023-02-13

**Authors:** Bin Wang, Kyungsook Kim, Mi Tian, Sumako Kameishi, Lili Zhuang, Teruo Okano, Yufeng Huang

**Affiliations:** 1 Department of Internal Medicine, Division of Nephrology & Hypertension, University of Utah Health Science, Salt Lake City, UT 84132, USA; 2 Cell Sheet Tissue Engineering Center (CSTEC), Department of Pharmaceutics and Pharmaceutical Chemistry, University of Utah Health Science, Salt Lake City, UT 84112, USA; 3 Institute of Advanced Biomedical Engineering and Science, Tokyo Women’s Medical University, Tokyo 162-8666, Japan

**Keywords:** MSC, cell sheet, regenerative therapy, chronic kidney disease

## Abstract

Although mesenchymal stem cell (MSC)-based regenerative therapy is being developed for the treatment of kidney diseases, cell delivery and engraftment still need to be improved. Cell sheet technology has been developed as a new cell delivery method, to recover cells as a sheet form retaining intrinsic cell adhesion proteins, which promotes its transplantation efficiency to the target tissue. We thus hypothesized that MSC sheets would therapeutically reduce kidney disease with high transplantation efficiency. When the chronic glomerulonephritis was induced by two injections of the anti-Thy 1.1 antibody (OX-7) in rats, the therapeutic efficacy of rat bone marrow stem cell (rBMSC) sheet transplantation was evaluated. The rBMSC-sheets were prepared using the temperature-responsive cell-culture surfaces and transplanted as patches onto the surface of two kidneys of each rat at 24 h after the first injection of OX-7. At 4 weeks, retention of the transplanted MSC-sheets was confirmed, and the animals with MSC-sheets showed significant reductions in proteinuria, glomerular staining for extracellular matrix protein, and renal production of TGFß1, PAI-1, collagen I, and fibronectin. The treatment also ameliorated podocyte and renal tubular injury, as evidenced by a reversal in the reductions of WT-1, podocin, and nephrin and by renal overexpression of KIM-1 and NGAL. Furthermore, the treatment enhanced gene expression of regenerative factors, and IL-10, Bcl-2, and HO-1 mRNA levels, but reduced TSP-1 levels, NF-kB, and NAPDH oxidase production in the kidney. These results strongly support our hypothesis that MSC-sheets facilitated MSC transplantation and function, and effectively retarded progressive renal fibrosis via paracrine actions on anti-cellular inflammation, oxidative stress, and apoptosis and promoted regeneration.

## 1. Introduction

Chronic mesangioproliferative glomerulonephritis (MsPGN) such as IgA nephropathy refers to a category of immunologically mediated chronic glomerular injury, which often leads to glomerulosclerosis and end-stage renal disease (ESRD). Currently, none of the available treatment agents can prevent, or even slow, the progression of MsPGN to ESRD. Novel targeted treatments are of pivotal clinical importance.

Mesenchymal Stem Cell (MSC)-based regenerative therapy has recently been highlighted as an alternative treatment strategy for many kidney diseases, including MsPGN [1]. It is well established that MSCs exhibit an excellent potential for accelerating tissue recovery or regeneration because of their self-renewal capacity, immunomodulatory effects, and ability to differentiate into various cell lineages. Many studies have demonstrated that MSCs could prevent renal injury and promote renal recovery by multiple mechanisms, such as immune modulation and paracrine factors, in animal models [1,2]. However, few MSC therapies have been approved, or are successful, although many clinical trials have been undertaken. MSC therapies are generally carried out by single cell injection, harvested from cultured MSCs using an enzymatic digestion such as trypsin-EDTA, or physical force, which leads to cells losing their inherent properties such as cell-cell junctions and extracellular matrix (ECM), often in unnatural settings. It is possible that a few functional MSCs would be present and act properly at the target sites through the cell injection delivery method [3,4]. 

To overcome the limitations of current MSC therapies, T. Okano pioneered and commercialized temperature-responsive cell culture surfaces [4,5]. The novel polymer-grafted tissue culture surface allows the release of cultured MSCs as confluent living cell sheets in response to small changes of the culture’s temperature (from 37 °C to room temperature), without any enzymatic digestion. Recovered cell sheets consisting of MSCs and their secreted matrix have several advantages over MSC suspensions or delivery of MSCs. MSC sheets completely maintain cells’ native settings and organization, including cell-cell communications, intact extracellular matrix (ECM) (the adherence protein layers), and tissue–like behaviors, compared with isolated MSCs in suspension, by using proteolytic enzymes [6,7,8]. Moreover, the intact ECM in MSC sheets also serves as a natural tissue adhesive that facilitates attachment to the targeted tissue surface without sutures. Using this technology, engineered living cell sheets have been developed and are now in human clinical trials for seven different organ areas (heart, cornea, esophagus, periodontal, middle chamber of ear, knee cartilage, and lung), in which cell sheets have shown a promising therapeutic effect in tissue regeneration [4,9,10,11,12,13,14]. We thus asked whether living cultured stem cell sheets, surgically applied to kidneys, could facilitate MSC transplantation and therapeutically inhibit kidney fibrosis through the paracrine and autocrine signaling pathways. To address these questions, we examined the effects and mechanisms of action of allogeneic bone marrow-derived MSC-sheets in a chronic model of rat mesangioproliferative glomerulonephritis (MsPGN) induced by two injections of monoclonal anti-Thy 1.1 antibody (OX-7) given one week apart, an established experimental model of progressive glomerulosclerosis.

## 2. Results

### 2.1. Characterization and Verification of Allogeneic MSC Sheets and Sheet Transplantation

**Figure 1:**
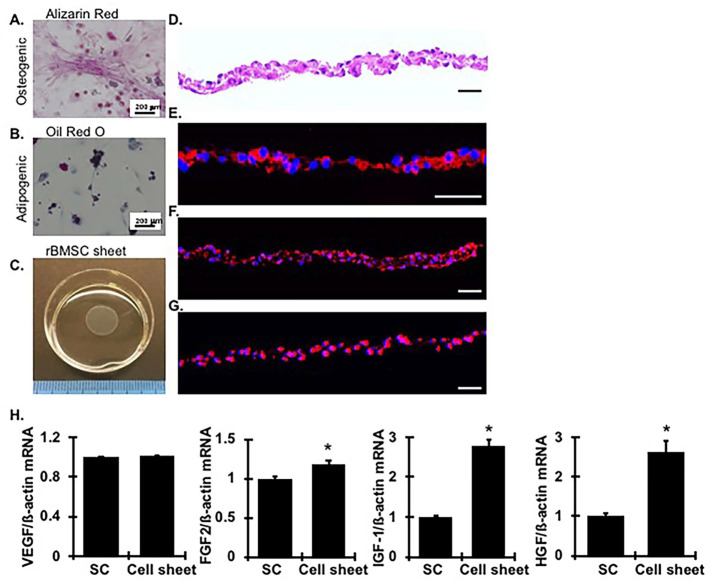
Characterization of rBMSC on the sheets. (**A**,**B**) rBMSCs obtained from SD rats were performed differentiation in adipogeneic and osteogeneic differentiation media for 3 weeks and stained with Alizarin-red (**A**) and Oil-red O (**B**), respectively. Scale bars represent 200 µm. (**C**) rBMSC sheet was detached from the culture dish by lowering the culture temperature. (**D**–**G**) Representative photomicrographs of an rBMSC sheet section with hematoxylin and eosin (H&E) staining (**D**) and immunofluorescent staining for HGF (in red) (**E**), FN (in red) (**F**) and ß-catenin (in red) (**G**). DAPI (in blue) stained nuclei served as cell counterstaining of the immunofluorescent staining. Scale bars represent 50 µm. (**H**) mRNA expression levels of VEGF, FGF2, IGF-1, and HGF in rBMSCs at the same number existed in single cell suspension (SC) or cell sheets. Measurements were carried out in triplicated dishes. * vs. SC, *p* < 0.05.

MSCs derived from rat bone marrow were positive for CD44 and CD90 (MSC markers) and negative for CD45 (hematopoietic marker). The MSC was able to undergo adipogenesis and osteogenesis, as can be verified by oil red O and alizarin red staining, respectively. As shown in Figure 1A, there were positive lipid droplets stained with oil red O, and calcium deposition stained with alizarin red, in the culture expanded MSC sheets, indicating adipogenesis and osteogenesis of the MSC sheets. A sample of the engineered rBMSC sheets is shown in Figure 1C. In the rBMSC sheet, all cells are connected each other and form a layer structure (Figure 1D). Positive expression of HGF, fibronectin, and ß-catenin was observed in the rBMSC sheet (Figure 1E–G).

Furthermore, when we identified the autocrine capability of MSC sheets, regenerative gene expression profiles of rBMSCs isolated from rBMSC sheets were determined and compared with those isolated from regularly passaged rBMSCs, and harvested through = trypsin-EDTA digestion. As shown in Figure 1H, rBMSCs present in cell sheets expressed all related regenerative genes including VEGF, FGF2, IGF-1, and HGF and the mRNA expression levels of those molecules, except VEGF, were higher than those in rBMSCs present in single cell suspensions (*p* < 0.05).

**Figure 2:**
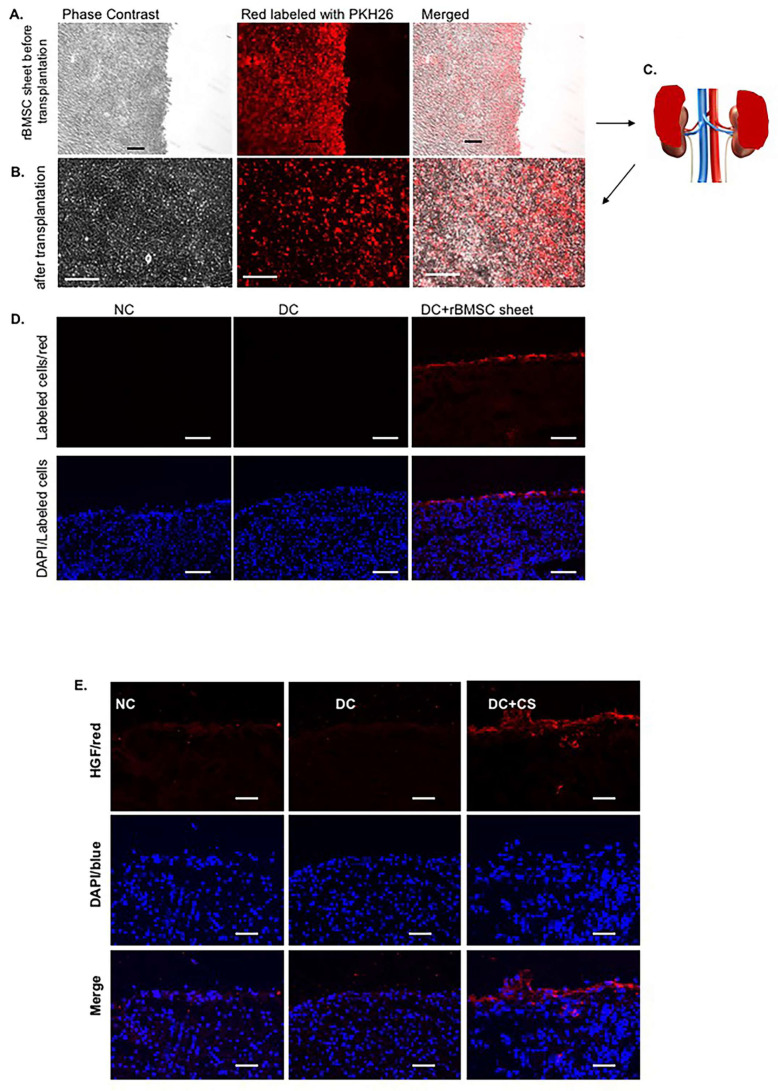
Verification of transplanted rBMSC sheets on the target site. (**A**,**B**) Representative photomicrographs of rBMSC sheets labeled with PKH-26 dye (in red) before transplantation (**A**) and after transplantation (**B**). Scale bars represent 100 µm. (**C**) Schematic representation of the transplantation sites of the rBMSC sheets in a rat with chronic glomerulonephritis. The renal capsule was removed from the anterior surface of the rat kidneys with tweezers, and one rBMSC sheet (in red) was placed on each site of the kidney where the renal capsule was removed. (**D**) Labeled rBMSC sheets with PKH-26 dye were observed directly on the surface of the frozen section of the kidneys at 4 weeks after transplantation. DAPI (in blue) stained nuclei served as cell counterstaining. Scale bars represent 100 µm. (**E**) Representative photomicrographs of immunofluorescent staining for HGF (in red) for rat kidneys from normal control (NC), untreated disease control (DC), and diseased rats that received rBMSC sheets (CS) transplantation (DC + CS). DAPI (in blue) stained nuclei served as cell counterstaining. Scale bars represent 50 µm.

In order to track the transplanted MSC sheets at the target site, we used a PKH26 red fluorescent cell linker kit to label the cell membranes on the MSC sheets, and strong red fluorescence was observed in the cells and sheets formed from rBMSCs, as shown in Figure 2A. The process of attaching cell sheets to the surface of the kidney is briefly shown as a cartoon picture in Figure 2C. Since the half-life for elution of PKH26 from the labeled cells is greater than 100 days, and the dye itself is stable and divided equally during cell proliferation, it is suitable for long term in vivo studies. As expected, MSC sheets with strong red fluorescence were observed directly when sheets were removed from the treated kidneys (Figure 2B), or kept on the cryosections of the treated kidney surfaces (Figure 2D) after MSC sheet transplantation for 4 weeks. No red fluorescence was observed in normal kidney capsules or disease control kidneys without rBMSC-sheet patches. These results confirmed the continued living attachment of the rBMSC sheets on the surface of the kidney in vivo. 

Of note, when we chose to stain the transplanted MSC sheets for HGF protein, to verify the paracrine function of rBMSCs on the sheets after transplantation for a long period of time, we found that HGF protein was stained not only in the whole rBMSC sheet but also in a few of the glomeruli and tubular cells in the kidney that received the rBMSC sheet transplantation. No obvious staining of HGF was found in the normal or diseased kidneys without the rBMSC sheet transplantation (Figure 2E). These results indicate that the transplanted rBMSCs on the cell sheets secrete paracrine factors continuously, and restore renal growth factor expression not only via paracrine secretion, but may also stimulate renal local resident cells to express and secrete growth factors, thereby promoting tissue regeneration and recovery.

### 2.2. Therapeutic Efficacy of rBMSC Sheets for Rat Chronic Glomerulonephritis

#### 2.2.1. Effect of rBMSC Sheets on Kidney Function, Proteinuria and Histology

The overall aim of this study was to assess the long-term effects of MSC sheets therapy in rats with chronic glomerulonephritis, compared to controls. Accordingly, animals were maintained at standard living conditions for 4 weeks after the induction of disease and treatment with rBMSC sheets, and renal function, blood pressure, body weight, and urinary protein excretion were determined. We prepared the three experimental groups including normal, untreated disease control but received sham surgery, and diseased rats treated with allogeneic bone marrow-derived MSC-sheets, and the final clinical parameters for the three groups of rats are shown in Table 1. All rats survived until the time of euthanasia. There was no difference in daily food intake and body weight among these three groups. All the rats with chronic glomerulonephritis still had normal blood pressure at 4 weeks after the disease induction. However, the diseased rats had increased serum BUN and creatinine (Cr) levels (*p* < 0.05) compared with the normal control rats. Treatment with rBMSC sheets for 4 weeks slightly but significantly reduced those measurements. The ratio of kidney weight to body weight in diseased rats was higher than that in normal rats, indicating kidney hypertrophy, which was ameliorated in cell sheets-treated diseased rats. 

**Figure 3:**
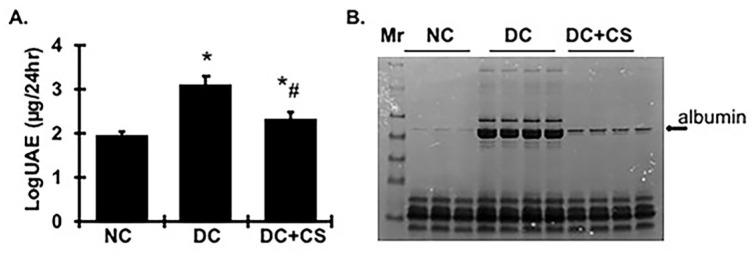
rBMSC sheets improve proteinuria in CGN rats. (**A**) log10-transformed urinary albumin excretion (UAE, µg/24 h) values. * vs. normal control rats (NC), *p* < 0.05; # vs. disease control rats DC, *p* < 0.05. DC + CS, diseased rats received rBMSC sheets transplantation. (**B**) SDS-PAGE electrophoresis of 24 h urine samples. Mr. protein markers.

Proteinuria is the hallmark of CKD and is an independent risk factor for renal disease progression. As shown in Figure 3A, urinary protein excretion increased significantly in diseased rats at 4 weeks after disease induction compared with normal rats, but was dramatically reduced in rBMSC sheets-treated diseased rats. These changes in urinary protein excretion are easily visualized by the markedly reduced amount of albumin protein in treated rats, shown on the electrophoresed SDS PAGE gel of urine samples in Figure 3B. 

**Figure 4:**
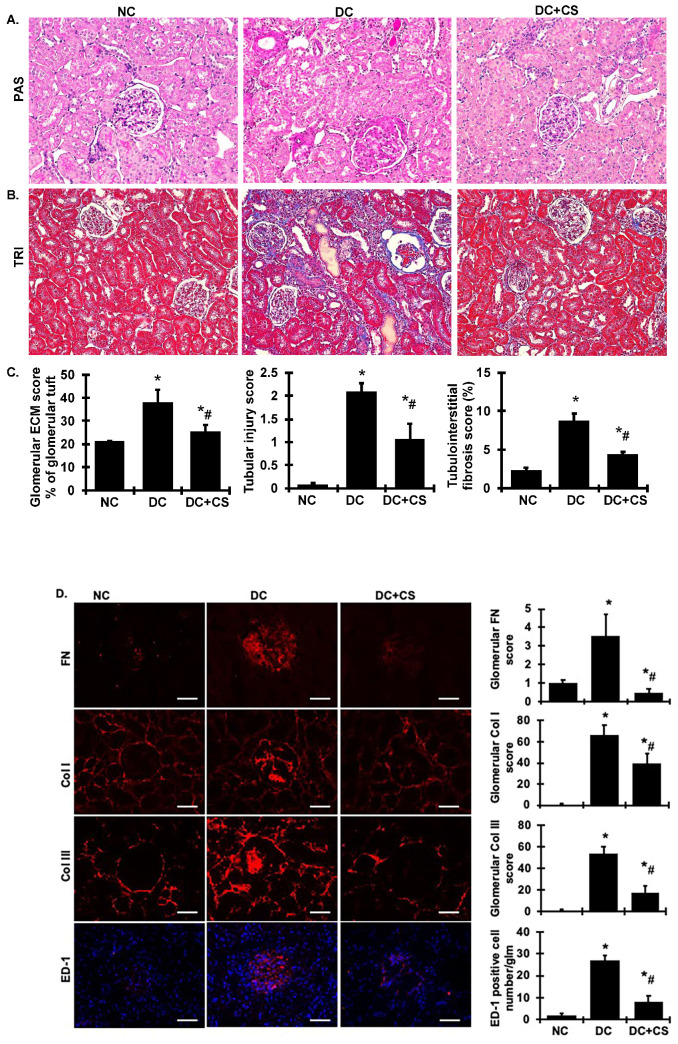
rBMSC sheets reduce glomerulosclerosis, tubular injury, tubulointerstitial fibrosis, and glomerular macrophages infiltration in CGN rats. (**A**) The histological renal sections stained with PAS showing glomerular ECM accumulation (stained pink) are presented at 200× magnification. (**B**) Masson’s trichrome (TRI) staining of the kidney sections used to detect tubular injury, inflammation, and collagen deposition were viewed with bright field illumination at 200× magnification. (**C**) The graphs summarize the results of the glomerular ECM staining score and tubular injury and tubular collagen deposition. (**D**) The immunofluorescent staining for renal sections for FN, Col I, Col III, and ED-1 positive macrophages. Scale bars represent 50 µM. Graphic representations of glomerular staining score or number of macrophages are shown on the right. * vs. normal control rats (NC), *p* < 0.05; # vs. disease control rats DC, *p* < 0.05. DC + CS, diseased rats received rBMSC sheets transplantation.

Histologically, untreated diseased rats developed glomerulosclerosis with a marked increase in the glomerular mesangial matrix, stained pink with PAS (Figure 4A), including matrix components measured by immunofluorescent staining for Col I, Col III, and FN (Figure 4D) at 4 weeks. Quantitative analyses of glomerular deposition of extracellular matrix (ECM) and matrix components are shown in Figure 4C,D. Focal tubulointerstitial lesions, determined by Masson’s Trichrome staining, were also present, composed of increased amounts of tubular disorder, collagen deposition (stained blue), and infiltrates of mononuclear cells in the untreated diseased rats (Figure 4B). The tubular injury score and tubulointerstitial fibrosis analysis are shown in Figure 4C. These lesions, in both the glomeruli and tubular areas, were markedly reduced in the rBMSC sheets-treated rats.

The number of monocytes/macrophages was determined in kidney sections from all rats in each group by the specific ED-1 antibody, that is known to label monocytes/macrophages. Nephritic glomeruli from untreated diseased rats contained higher numbers of monocytes/macrophages positive expression areas than did glomeruli from the normal rats, indicating an accumulation of macrophages and inflammation in the diseased kidney tissue. However, ED-1+ macrophages were reduced in the kidney tissue in the rBMSC sheets-treated rats compared with the untreated diseased rats (Figure 4D). Of note, no ED-1+ macrophages were observed in any rBMSC sheet areas.

#### 2.2.2. Effect of rBMSC Sheets on Renal Fibrotic Markers

**Figure 5:**
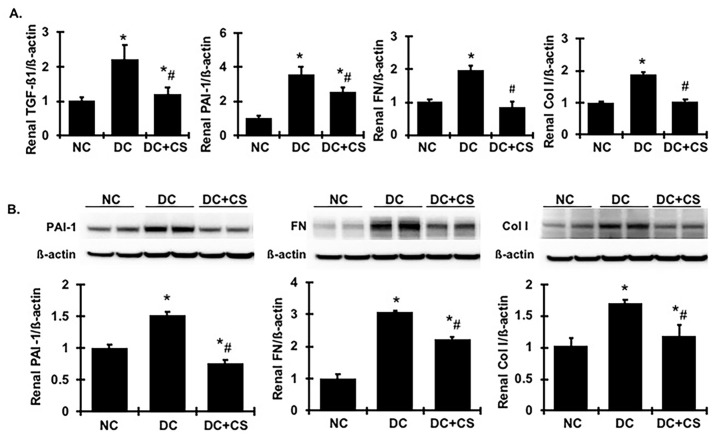
rBMSC sheets reduce renal mRNA expression and protein production of profibrotic markers in CGN rats. (**A**) Expression of TGFß1, PAI-1, FN, and Col I mRNA. Expression of mRNA was determined by real-time RT/PCR. Changes in mRNA levels were determined by first correcting the amplification of ß-actin for each sample. (**B**) Representative Western blots illustrating PAI-1, FN, Col I, and ß-actin protein expression in renal cortical tissues. The graphs summarize the results of band density measurements for PAI-1, FN, and Col I. * vs. normal control rats (NC), *p* < 0.05; # vs. disease control rats DC, *p* < 0.05. DC + CS, diseased rats received rBMSC sheets transplantation.

As two key modulators of matrix accumulation, TGFß1 and PAI-1 play an important role in renal fibrosis. As expected (Figure 5A), renal TGFß1 and PAI-1 mRNA levels were increased by 2.23-fold and 3.59-fold, respectively, in the untreated diseased rats, compared with those of their normal control littermates. Consequently, renal FN and Col I mRNA levels were increased in diseased rats (Figure 5A). In agreement with the increased mRNA expression levels, the protein levels of PAI-1, FN, and Col I in the renal cortex tissue, further quantified by Western blot assay, were markedly increased in the diseased rats, compared with the normal rats (Figure 5B). However, both the mRNA expression and the protein production levels of these fibrotic markers in the renal cortex induced by chronic glomerulonephritis seen in untreated rats were decreased significantly in the rBMSC sheets-treated rats. 

#### 2.2.3. Effect of rBMSC Sheets on Podocyte Injury

Although the primary injury, i.e., injection of a complement-activating anti-Thy 1.1 antibody, OX-7, is highly selective for renal mesangial cells, intraglomerular capillaries are destabilized in the course of mesangial cell destruction, leading to capillary dilation and intraglomerular microaneurysms after disease induction [15]. These changes contribute to secondary podocyte damage via altered physical forces and/or potential biochemical alterations of the glomerular basement membrane [16,17], which may promote proteinuria and ultimately glomerular sclerosis in anti-Thy 1.1 nephritis.

**Figure 6:**
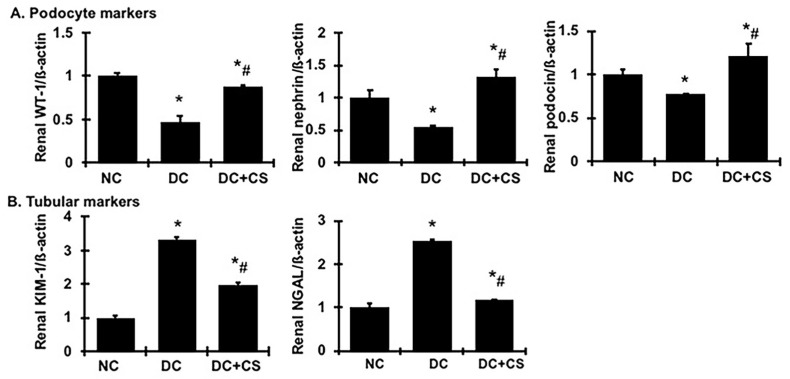
rBMSC sheets ameliorate mRNA expression of podocyte markers and tubular injury markers in CGN rats. (**A**) Expression of WT-1, nephrin, and podocin mRNA in renal cortex tissue. (**B**) Expression of KIM-1 and NGAL mRNA in renal cortex tissue. Expression of mRNA was determined by real-time RT/PCR. Changes in mRNA levels were determined by first correcting the amplification of ß-actin for each sample. * vs. normal control rats (NC), *p* < 0.05; # vs. disease control rats DC, *p* < 0.05. DC + CS, diseased rats received rBMSC sheets transplantation.

As shown in Figure 6A, detected by real time RT/PCR, the mRNA expression of WT-1, podocin, and nephrin was down-regulated substantially in this model (Figure 6A). Treatment with rBMSC sheets largely preserved the expression of these podocyte markers. Although we did not investigate the changes of podocyte morphology, these data suggest that treatment with rBMSC sheets may ameliorate podocyte injury and loss in this model, thereby resulting in a significant decrease in proteinuria and remission of renal fibrosis.

#### 2.2.4. Effect of rBMSC Sheets on Renal Tubular Injury 

Renal tubulointerstitial damage is known to be the final common pathway of all progressive chronic kidney diseases [18]. In the rat chronic glomerulonephritis model, renal tubular cells can be injured by reabsorbing increased amounts of protein from the tubular lumen. This may lead to inflammation and fibrosis in the tubulointerstitial area, as we observed in the untreated diseased rats (Figure 4B). Thus, the effect of rBMSC sheets on the improvement of tubule-interstitial injury was further assessed. 

Both the kidney injury molecule-1 (KIM-1) and neutrophil gelatinase-associated lipocalin (NGAL) have been validated as early predictive markers of renal tubular injury, and their sustained expression is usually correlated with the extent of renal tubular inflammation and fibrosis [19,20]. As shown in Figure 6B, renal KIM-1 and NGAL were highly expressed in the diseased rats compared to the normal rats. Surprisingly, both of them in the rBMSC sheets-treated rats were much lower than those in the untreated diseased rats, by 59% and 89%, respectively, although they were still higher than those in the normal rats. These results indicate that tubular injury had occurred in chronic glomerulonephritic rats but was much less in the rBMSC sheets-treated nephritic rats.

#### 2.2.5. Effect of rBMSC Sheets on Cellular Signaling Pathways Involved in the Progression of CKD

**Figure 7:**
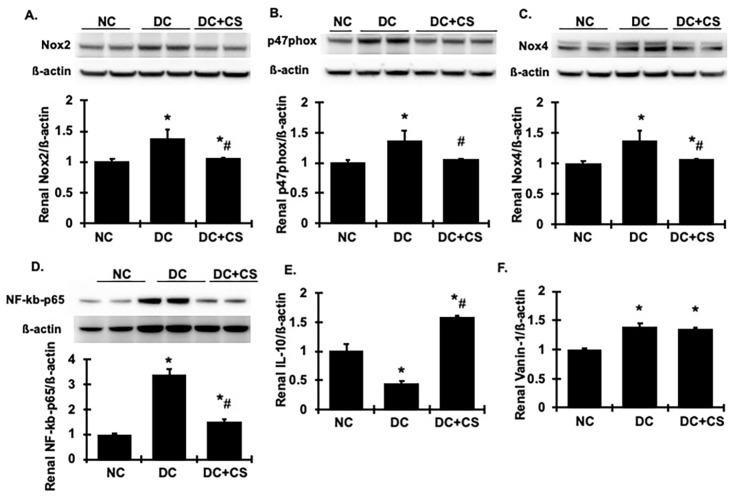
rBMSC sheets reduces renal oxidative stress and related renal information markers in CGN rats. (**A**–**D**) Representative Western blots illustrating Nox2 (**A**), p47^phox^ (**B**), Nox4 (**C**), NF-kB-p65 (**D**), and ß-actin protein expression in renal cortical tissues. The graphs summarize the results of band density measurements shown under the blots. (**E**,**F**) Expression of IL-10 and Vanin-1 mRNA in renal cortex tissue. Expression of mRNA was determined by real-time RT/PCR. Changes in mRNA levels were determined by first correcting the amplification of ß-actin for each sample. * vs. normal control rats (NC), *p* < 0.05; # vs. disease control rats DC, *p* < 0.05. DC + CS, diseased rats received rBMSC sheets transplantation.

Based on the previous findings of MSC-based regenerative therapy [1], the potential protective strategies of rBMSC sheets may include their capacity to interfere with the inflammation process, mainly with the action of chemokines, cytokines, or macrophages infiltration, the capacity to interfere with programmed cell death (apoptosis), and the capacity to interfere with the renal regeneration process. Inflammation is the main physiopathology of CKD. We tested macrophage infiltration in the injured kidney (Figure 4B,D). We then tested renal phosphorylated NF-kB-p65 protein production by Western blot analysis, and the results revealed an increased renal production of p-NF-kB-p65 in the diseased rats compared with the normal rats, which was lowered significantly by the treatment with rBMSC sheets (Figure 7D). Furthermore, renal protein production of Nox2 and p47^phox^, and Nox4, the family members of NAPDH oxidase, was much greater in the diseased rats compared with the normal controls (Figure 7A–C), indicating significant renal activation of Nox2 and Nox4 in these diseased rats. However, both of them were largely reduced in the rBMSC sheets-treated rats, approaching normal levels observed in uninjured kidneys (Figure 7A–C). Together, these data indicate that treatment with rBMSC sheets inhibits NF-kB-mediated inflammation and NAPDH-mediated oxidase generation and activation in the kidney with chronic glomerulonephritis. 

Interleukin 10 (IL-10), as a potent anti-inflammatory cytokine, plays a crucial and often essential role in preventing inflammatory and autoimmune pathologies through curbing the production of pro-inflammatory molecules to limit tissue damage and to maintain or restore tissue homeostasis [21]. In our model, the diseased rats had a markedly reduced expression of IL-10 in the kidney. However, renal IL-10 was highly expressed in rBMSC sheet-treated animals, and it was even higher than that in normal kidneys (Figure 7E). Vanin-1 is an epithelial ectoenzyme which activates the conversion of pantetheine into pantothenic acid (vitamin B5) and cysteamine [22], the latter has been found to inhibit the activities of the antioxidant superoxide dismutase and glutathione, allowing free radicals to promote oxidative tissue damage [23,24]. In this study, the upregulation of vanin-1 in the chronic glomerulonephritic rats was also observed in diseased kidneys. However, treatment with rBMSC sheets failed to alter the overexpression of vanin-1 seen in the diseased kidneys (Figure 7F). Together, these results revealed that the rBMSC sheets-treated rats had fewer macrophages infiltrating the kidney tissue, which lowered the expression of inflammatory cytokines in combination with increased expression of anti-inflammatory factors, which are linked to reduced renal fibrosis. 

**Figure 8:**
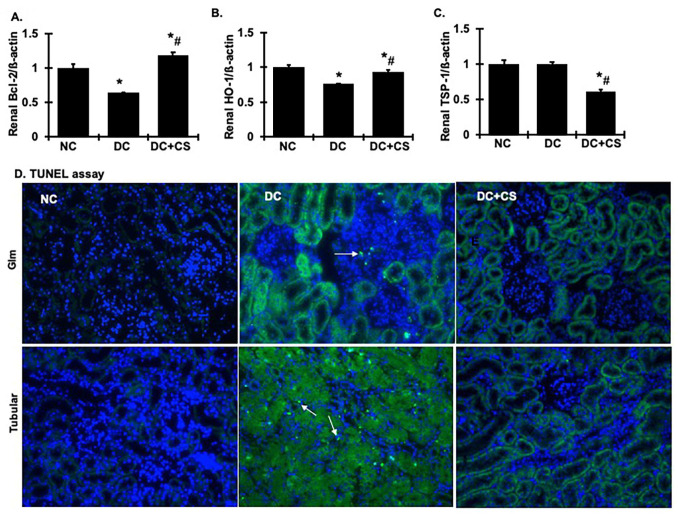
rBMSC sheets ameliorate apoptosis related mRNA expression and TUNEL-positive cells in renal cortex tissue of CGN rats. (**A**–**C**) Expression of Bcl-2, HO-1, and TSP-1 mRNA in renal cortex tissue. Expression of mRNA was determined by real-time RT/PCR. Changes in mRNA levels were determined by first correcting the amplification of ß-actin for each sample. (**D**) Representative micrographs of TUNEL staining at 200× magnification for glomeruli (Glm) and tubulointerstitial area (Tubular). DAPI (in blue) stained nuclei served as cell counterstaining. White arrows indicate TUNEL-positive cells. Analysis of renal TUNEL-positive number of cells was included in the text (NC, 0.152 ± 0.152; DC, 28.37 ± 8.57, and DC + CS, 3.453 ± 1.643, per 1000 cells). * vs. normal control rats (NC), *p* < 0.05; # vs. disease control rats DC, *p* < 0.05. DC + CS, diseased rats received rBMSC sheets transplantation.

In addition, we briefly scanned the role of apoptosis in our model by quantifying anti-and pro-apoptotic genes such as HO-1, Bcl2, and TSP-1 in the kidney tissue. It was found that mRNA levels of anti-apoptotic genes, such as HO-1 and Bcl2, in the kidney were decreased in the untreated diseased rats compared with the normal animals, and were largely restored in the rBMSC sheet-treated animals (Figure 8A,B). In contrast, although there were no changes in TSP-1 mRNA levels in the untreated diseased or normal rats, TSP-1 mRNA levels in the kidney were reduced in the rBMSC sheet-treated rats (Figure 8C). TSP-1 is known to be transactivated by p53 and causes apoptosis in cultured kidney cells; TSP-1 null mice are protected against kidney failure and structural damage in ischemia/reperfusion injury model [25]. Our finding suggests that, in contrast to the normal control groups, the expression pattern of mRNA of anti- and pro-apoptotic genes in diseased rats was observed toward cell death, while treatment with rBMSC sheets restored the situation toward normalcy. To further support our findings, we screened renal tissue for TUNEL-positive cells and observed that there was a significant increase in TUNEL-positive cells in both the renal glomerular and tubulointerstitial areas in diseased rats in comparison to the normal controls (the TUNEL-positive cell number in diseased kidneys was 28.37 ± 8.57 per 1000 cells vs. 0.152 ± 0.152, per 1000 cells in normal kidneys, *p* < 0.05), as shown in Figure 8D (indicated by white arrows). There was no significant apoptosis of renal cells in the rBMSC sheets-treated rats (The TUNEL-positive cell number was 3.453 ± 1.643, per 1000 cells, vs. NC, *p* < 0.05; vs. DC, *p* < 0.05) indicating that re-balancing the anti- and pro-apoptotic gene expressions by rBMSC sheets is contributing significantly to inhibition of the apoptosis of renal cells and injury. 

**Figure 9:**
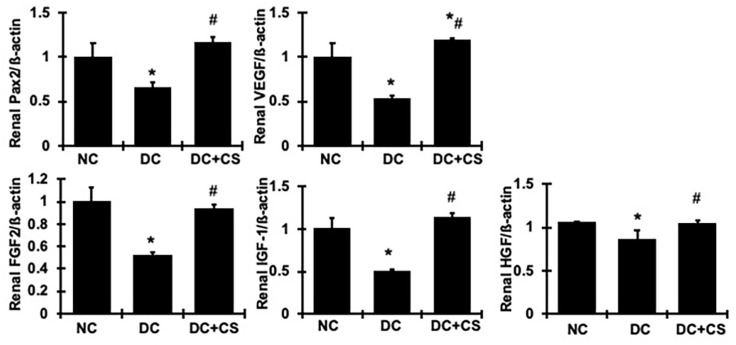
rBMSC sheets ameliorate mRNA expression of regenerative factors in renal cortex tissue of CGN rats. Expression of mRNA was determined by real-time RT/PCR. Changes in mRNA levels were determined by first correcting the amplification of ß-actin for each sample. * vs. normal control rats (NC), *p* < 0.05; # vs. disease control rats DC, *p* < 0.05. DC + CS, diseased rats received rBMSC sheets transplantation.

The Pax2 gene encodes a DNA-binding transcription factor whose expression is related to cell regeneration [26]. In the rBMSC sheet-treated animals, there was an increase in Pax2 mRNA expression in the kidney compared with the decreased expression of Pax2 in the untreated animals. To further verify this increase, we quantified the common molecules involved in the process of renal regeneration: VEGF, FGF2, HGF, and IGF-1 [27]. Again, the rBMSC sheets-treated animals showed a higher gene expression level in these regenerative factors in the kidney, while all of them were reduced in the untreated diseased rats (Figure 9). These measurements only represented the treated kidneys and did not include the growth factor levels generated by the rBMSC sheet itself, since the rBMSC sheet was removed when the kidney was harvested. The increased extent of these growth factors is likely much less than the total amount of growth factors that may affect the treated kidneys. Nonetheless, these results indicate that the functional rBMSC sheets may also stimulate renal local resident cells to express and secrete regenerative growth factors thereby promoting tissue regeneration and recovery.

## 3. Discussion

Anti-Thy 1.1 nephritis is a well-characterized experimental model of immune complex-mediated mesangioproliferative glomerulonephritis (MsPGN). It has been shown that OX-7 binds to a Thy 1.1 epitope on the surface of mesangial cells and causes complement-dependent cell lysis followed by exuberant matrix synthesis and deposition, which mimics an antigen-triggered immune mesangial proliferative glomerulonephritis in humans. In particular, two episodes of glomerular immune injury, induced by injections of OX-7 mAb given one week apart, are reported to produce an irreversible chronic model of anti-Thy 1.1 nephritis [28], which mimics continued immune injury, as occurs in lupus nephritis or IgA nephropathy in humans, producing chronic disease and kidney failure. This is a valuable model for evaluating the effects of interventions on progressive proteinuria, impaired renal function, and glomerular and tubular fibroses. 

In the present study, we have shown that two repeated injections of the OX-7 antibody produce progressive glomerulonephritis in rats, characterized by elevated levels of urinary protein and blood urea nitrogen (BUN) and creatinine (Cr). Accompanying the functional changes is the persistence of podocyte injury, pathological glomerular ECM expansion, and matrix components (FN and collagen) deposition, glomerular infiltration of circulating inflammatory cells, tubular injury, tubulointerstitial fibrotic morphology, increased mRNA expression and protein production of pro-inflammatory and oxidative stress-related and profibrotic molecules, and increased apoptosis and decreased tissue repair capability. When disease severity is determined in the chronic model of anti-Thy 1.1 nephritis [28], there has been no marked difference found in this model with or without decapsulating both kidneys for 4 weeks. Of note, when allogeneic bone-marrow-derived stem cell sheets were patched on the surface of the diseased kidney in this model, for 4 weeks, the continued living attachment of the rBMSC sheets with living functional rBMSCs on the surfaces of the kidneys was confirmed, and these rats showed reduced disease severity, as evidenced by ameliorated proteinuria, renal functions, and renal structural changes that occurred in the disease control rats. Our data clearly indicate that rBMSC sheets are effective for inhibition of the progression of proteinuria and glomerular and tubular injury associated with MsPGN. 

Consistent with the established features of MSCs, our data indicate that the rBMSCs maintained on the sheets likely secrete several cytokines and growth factors that modulate inflammation, oxidative stress, apoptosis, and regenerative pathways in the adjacent kidney cells, thereby decreasing the progression of the kidney disease. In addition, we observed that the diseased kidneys covered with rBMSC sheets also expressed more trophic factors, which might facilitate endogenous renal regeneration as well. Together with the recent report that conditional medium derived from stem cells was able to improve renal function [29], our study suggests that paracrine effects by trophic factors released from MSCs are important for kidney repair. Recent studies further revealed that MSCs release extracellular vesicles, or exosomes, that carry a combination of mRNA and microRNA capable of regulating the transcription of genetic information and modulating angiogenesis, inflammation, and so on in recipient cells [30,31,32]. Whether such an exosome-mediated mechanism is relevant to the action of rBMSC sheets in this study needs to be further determined. In addition, the immunosuppressive properties of MSCs has been reported in a great number of studies, including inhibition of the proliferation of CD8^+^ and CD4^+^ T lymphocytes and natural killer cells, inhibition of maturation of dendritic cells, and stimulation of the proliferation of regulatory T cells [33]. It is possible that such an immunomodulatory property of rBMSCs may reduce the repeated episodes of immune injury in the present animal model when rats received the second injection of OX-7. Nonetheless, the paracrine effects of rBMSC sheets may serve as the possible main mechanism to protect the kidney cell against inflammation, NAPDH-mediated oxidative stress and apoptosis, and promote kidney cell regeneration, thereby slowing the progression of injury to ESRD, as summarized in the graphical abstract. Similar protective effects of stem cell sheets have been reported in other models for myocardial infarction, dilated cardiomyopathy, full-thickness skin wounds, hind limb ischemia, esophageal strictures, and oral ulcers [4,9,10,11,12,13,14]. To our knowledge, the present study is the first time that the therapeutic potential of stem cell sheets for progressive glomerulosclerosis has been shown. However, it is unknown whether the rBMSCs on the sheet are able to migrate into glomeruli and/or tubular areas to participate in the regeneration of kidney cells directly. We plan to transplant GFP-rBMSC sheets to track the transplanted cells for a long-term period in the next experiments.

MSCs, or related cells, have been shown to produce beneficial effects in animal models for a variety of kidney diseases including acute or chronic renal injury, caused by ischemia reperfusion injury alone or in combination with the administration of gentamicin, 5/6 nephrectomy, unilateral ligation, a one-shot injection of the anti-Thy 1.1 antibody followed by unilateral nephrectomy, or even type 1 diabetes, when exogenous MSCs are transplanted via the tail vein, or artery, intracardiac, or renal artery injection [33,34]. The common phenomena observed from those studies are that a few injected MSCs are able to home to the injury site, particularly the glomerulus region or tubular area, and that the recruited MSCs in the kidney also disappear quickly. Such low efficiency of cell engrafting and function, may correlate with cell isolation using a traditional enzymatic digestion, which causes cells to lose their inherent properties and function, as discussed in the introduction. It also may mainly correlate with the cell delivery method. For example, following i.v. infusion, the majority of the cells may be trapped in the capillary beds of the lung, without playing a substantial role in the kidney. While increasing the number of administered MSCs to compensate for this disadvantage may cause unwanted side effects, such as embolism. Arterial or direct renal arterial administration of MSCs seems be more effective than systemic intravenous administration in homing cells to the kidney. However, the specific kidney structure causes most infused cells to be distributed throughout the glomerular area of the kidney [35]. It has been shown that high numbers of glomeruli of MSC treated rats were observed to be differentiated into fat cells, which obstructed the affected nephrons and led to periglomerular fibrotic response, when MSCs were given via direct renal arterial injection [36]. When MSCs were infused into the suprarenal aorta, no adipogenic differentiation of MSCs was observed, while most of MSCs were found to not remain in the kidney for longer than 3 days [37]. Such findings indicate that infused MSCs found in the kidney at the optimal dose, even for a short time, seem to be sufficient to elicit a temporary protective response in animal models, while MSCs may not be able to per se contribute to subsequent renal repair for a long term. It is unknown whether these limitations are the reason why few clinical MSC therapies have been successful despite many clinical trials being undertaken.

Are these MSCs maintained on cell sheets able to overcome the limitations of current MSC therapies and function better for kidney repair? To address this question, we actually included a diseased group treated with a classic MSC suspension in this study, in which the same number of rBMSCs (6 × 10^4^ rBMSCs) as a cell suspension, as counted on two cell sheets, was injected into diseased rats (*n* = 7) at day 1, after the first OX-7 injection, through the left carotid artery. Those rats were managed and maintained in the same way as the rBMSC sheet-treated group. Unfortunately, this cell suspension treatment for 4 weeks had no effect on proteinuria and glomerulosclerosis in the present model, and renal fibrotic and cellular injury markers were not ameliorated by this treatment either (see Supplementary Tables S2 and S3). These results are inconsistent with the protective effect of MSCs observed previously for progressive glomerulosclerosis [36,38]. We consider that there are at least two possible reasons for these inconsistencies. The first, maybe the main reason, is that previous studies have used a much higher number of MSCs (Dr. Oite group used 1 × 10^8^ of MSCs via i.v. injection and Dr. Floege group used 2 × 10^6^ of MSCs via renal artery injection.) than we used (6 × 10^4^ rBMSCs) for rats. Another possibility is that they used a different chronic glomerulonephritis model, induced by a one-shot injection of the anti-Thy 1.1 antibody combined with uninephrectomy, which makes the number of MSCs acting on one kidney much higher than the two kidneys used in the present study. On the other hand, these results may suggest that rBMSCs maintained as a living cell sheet actually function much better and longer than as a cell suspension, and even with a lower number of rBMSCs on a sheet, this is sufficient to limit the progression of glomerulosclerosis. Thus, rBMSC sheets may treat renal fibrosis effectively by their enhanced therapeutic capabilities, compared to common injected cell suspension therapies. 

It has been shown that MSC treatment is safe and well tolerated. However, concerns have also been raised that MSCs infusion may have the potential risk of tumorigenicity or unwanted tissue formation [39]. In the present study, we observed that the rBMSC sheets retained over 95% of transplanted cells on the surfaces of targeted kidneys for 4 weeks. No unwanted tissue formation was observed yet on the surface of the targeted kidneys. In addition, treatment with MSC sheets should reduce the risk of complications induced by injected cell migration into non-targeted tissues. Nonetheless, the long-term effects of MSC sheets in vivo, such as, cell survivability, migration, function, and so on, still needs to be further determined. 

In conclusion, allogeneic rBMSC sheets applied on the surfaces of kidneys exhibited significantly lower host immunoreactions and promoted recovery from chronic glomerulonephritis in a rat model, based on assessments of functional and structural aspects. The ability of the rBMSC sheets to attenuate the progression of chronic glomerulonephritis is possibly due to their paracrine effects caused by trophic factors released from the rBMSCs. Together with the reality that rBMSC sheets can be easily delivered to patients’ kidney capsules by using a special needle, through a similar process of kidney biopsy, under the guidance of an imaging device, these results suggest that MSC sheets should be further explored as a promising clinical treatment approach to improve the therapy for renal fibrosis for patients with CKD, or to improve outcomes for renal transplantation by protecting donor kidneys from ischemia-reperfusion-induced fibrosis and transplant injury or host immunological responses. 

## 4. Methods and Materials

### 4.1. Reagents

Unless otherwise indicated, all materials and chemicals were obtained from Sigma-Aldrich (St. Louis, MO, USA).

### 4.2. Animals

All studies were performed in male Sprague–Dawley rats obtained from Charles River. Animal housing and care were in accordance with the NIH Guide for the Care and Use of Laboratory animals. All procedures involving animals described herein were approved by the Animal Care Committee of the University of Utah. 

### 4.3. Fabrication of Mesenchymal Stem Cell Sheets

The rat bone marrow derived-stem cells (rBMSCs) were from the same species of rats (Sprague–Dawley rats, weighing 100 g), with the disease model for all experiments being generated by standard procedures. In brief, femurs of sacrificed rats were flushed with phosphate buffered saline (PBS), and bone marrow cells were collected in tubes, washed, and placed in plastic Falcon tissue culture flasks with DMEM medium supplemented with 10% fetal bovine serum (FBS) and 100 U/mL penicillin/streptomycin in a 37 °C, 5% CO_2_ incubator. After five days of culture, non-adherent cells were removed and attached MSCs were harvested, used differentiation potential test and fabrication of stem cell sheets.

The characterized rBMSCs at passage five were seeded and cultured on temperature-responsive cell culture dishes (TRCDs) to form rBMSC sheets with DMEM medium mixed with 20% FBS and 100 U/mL penicillin/streptomycin in a 37 °C, 5%CO_2_ incubator for five to six days, and 16.4 µg/mL ascorbic acid was added into the medium at day 1. The sheets were transferred to room temperature for an hour and then were spontaneously detached from the culture dishes. The TRCDs are hydrophobic at 37 °C, but become hydrophilic at room temperature (20 °C to 24 °C), which allows the cell sheets to detach from the dishes while maintaining their cell-cell junctions and adhesion molecules. The structural integrity of rBMSC sheets were confirmed by hematoxylin and eosin staining and immunofluorescent staining for intrinsic extracellular matrix (fibronectin; Abcam, Cambridge, MA, USA), cell junction (ß-catenin; Abcam) and hepatocyte growth factor (HGF, Abcam). To determine the autocrine capability of the MSC sheets, regenerative gene expression profiles of rBMSCs isolated from rBMSC sheets or regularly passaged rBMSCs were detected by real time RT/PCR and normalized by expression of house-keeping gene as described below. Further, the rBMSC sheets were labeled with PKH26 by using the PKH26 red fluorescent cell linker kit before transplantation, according to the manufacturer’s instructions. The labeled cell sheets were attached to CellShifter^TM^ membranes (Thermo Fisher Scientific, Waltham, MA, USA) that were then transplanted onto the decapsulated surfaces of kidneys in an hour. 

### 4.4. Induction of Chronic Glomerulonephritis

Twenty-one male Sprague–Dawley rats, weighing 180 to 200 g, were divided into three groups of seven: normal control, untreated disease control, and allogeneic rBMSC-sheets treated diseased rats. Chronic mesangioproliferative glomerulonephritis (MsPG) was induced in groups 2 and 3 rats by two-shot tail vein injections of the monoclonal anti-Thy 1.1 antibody (Ab) OX-7 (1.875 mg/kg body weight) on day 0 and day 7. The OX-7 mAb was produced by cultured OX-7 cells, as described previously [40]. The normal control animals were injected with the same volume of PBS. Treatment was started one day after the first injection of OX-7 mAb.

### 4.5. Surgical Procedures and rBMSC Sheet Treatment

Animals were anesthetized with isoflurane. The right kidney was founded and decapsulated on the abdominal side. The rBMSC sheet (0.7 cm × 0.7 cm with 3 × 10^4^ rBMSCs) was applied on the decapsulated surface side of the right kidney. After 10 min, which was demonstrated to be a sufficient time for the cell sheet to attach to the surface of kidney, the shifter was removed, leaving behind only the cell sheet. During the incubation time, 1 mL sterilized PBS in drops was continuously applied on the surface of the rBMSC sheet to facilitate the sheet to attach the kidney. Then all organs were relocated in their own places. The same procedure was performed on the left kidney. After rBMSC sheets overlaid on the surface of both kidneys, the abdominal surgical incision was cleaned and sutured closed. Untreated disease rats received sham surgery by decapsulating both kidneys served as surgical control. The experiment was terminated 4 weeks after treatment. Body weight, 24-h water intake and 24-h urine samples for the measurement of albumin were collected from each rat in individual metabolic cages every week after treatment. Urine albumin was measured using the DC 2000 + microalbumin/creatinine reagent kit (Bayer HealthCare, Elkhart, IN, USA). 

### 4.6. Euthanasia

Rats were euthanized under isoflurane anesthesia, after treatment for 4 weeks. The rBMSC sheets were observed covering both kidneys in the treated rats. Mean arterial pressure (MAP) was recorded by the Digi-Med Blood Pressure Analyzer^TM^ (BPA-400, Micro-Med, Inc., Louisville, KY, USA) via lower abdominal aorta catheterization with heparin-filled pressure transducers (25 IU/mL). Five to 10 mL blood was then drawn from the lower abdominal aorta and the kidneys were perfused with 60 mL ice-cold PBS. One piece of renal cortical tissue (with the rBMSC sheets in treated rats) was fixed in 10% neutral-buffered formalin for histological examination. One piece of renal cortical tissue (with the rBMSC sheet in treated rats) was snap frozen for frozen sectioning. The rBMSC sheet on the left part of renal cortex in treated rats was removed and put in cold PBS for histology analysis, while the normal renal capsule was also removed from normal control rats and served as the control. Other pieces of renal cortex tissue were harvested by dissection and stored in liquid nitrogen for Western blot or treated with Tri Reagent for isolation of RNA assay kit (PIERCE, Rockford, IL, USA). 

### 4.7. Determination of Renal Function

Serum BUN concentrations were measured by using the QuantiChrom^TM^ urea assay kit with a modification by using 15 µL samples or standard (BioAssay System, Hayward, CA, USA). Serum creatinine (Cr) levels were determined by using the rat creatinine assay kit (Crystal Chem, Elk Grove Village, IL, USA).

### 4.8. Histological Analysis

Three-micrometer sections of paraffin-embedded kidney tissues were stained with Periodic Acid-Schiff (PAS) and Masson’s Trichrome (TRI) by the histology core facility at the University of Utah and scored in a blinded fashion, with a semi-quantitative ordinal scale, as described previously [40,41]. Briefly, glomerular extracellular matrix (ECM) content (stained pink with PAS) was calculated as the fraction of glomerular matrix area to the total glomerular tuft area in pixels by using the Image-J software. Masson’s trichrome staining of the kidney sections was used to detect tubular injury, inflammation and collagen deposition. The tubular features such as brush border loss, dilatation, intraluminal cast, disappeared physical back to back position with enlarged spaces in between, interstitial inflammatory cell infiltration, and epithelial cell apoptosis were quantified as tubular injury. Ten non-overlapping fields of renal tubulointerstitial area were scored with a semi-quantitative ordinal scale (grade 0 for tubular injury % ≤5%; grade 1 for >5% to ≤25%; grade 2 for >25% to ≤50%; and grade 3 for >50%) and the mean used as the injury score. Tubulointerstitial collagen deposition (stained blue) was quantified by the Image-J software as the percentage of the detection area in pixels.

Immunofluorescent staining for fibronectin-EDA+ (FN), type I collagen (Col I), type III collagen (Col III), HGF, and macrophages was performed on frozen renal sections and evaluated in 20 glomeruli from each rat, as described in [40]. Briefly, samples were fixed with 4% paraformaldehyde (PFA) for 10 min and permeabilized with 0.1% triton X-100 (Thermo Fisher Scientific) for 10 min. Non-specific binding was blocked in PBS containing 10% serum (Vector Laboratories, Burlingame, CA, USA), for 1 h at room temperature. Primary antibodies of mouse anti-fibronectin-EDA+ (FN) IgG (Harlan Sera-Lab Ltd., Loughborough, United Kingdom), goat anti-type I collagen (Col I), goat anti-type III collagen (Col III) IgG (Southern Biotechnology Associates, Birmingham, AL, USA), mouse monoclonal anti-CD68 (ED-1) antibody (Abcam), and rabbit anti-HGF IgG (Abcam) were applied at 4 °C overnight. Either Alexa Fluor 594-conjugated antibody (Life Technologies, Carlsbad, CA, USA) or rhodamin conjugated fragment anti-goat IgG (Jackson Immuno Research Labs, West Grove, PA, USA) was used as the secondary antibody. Intraglomerular positive staining of FN, Col 1, Col III, and ED-1 was quantified separately, in a blinded fashion, using the Image-J software (National Institutes of Health, Bethesda, MD, USA) as described previously [40,41]. 

### 4.9. TUNEL Labeling of Renal Cell DNA for Detection of Apoptosis

The TUNEL assay, that is most often used to identify apoptosis [42], was performed on formalin-fixed renal cortex tissues using the In Situ Cell Death Detection Kit (Roche) following the manufacturer’s instructions. After staining, one drop of DAPI-Fluoromount-G was applied on the section to stain the nuclei. The TUNEL-positive cells in the renal tissue were quantified in 5 randomly selected areas per section, under ×200 magnification, by calculating the percentage of the total cell number of the section using the ImageJ software.

### 4.10. Western Blot Analysis

Kidney protein was isolated from kidney cortex tissue (15 mg) from each rat, pooled at an equal amount in each group to represent the individual group, and then immunoblotted in duplicate on immobilon-p transfer membranes (Thermo Fisher Scientific). Proteins of plasminogen activator inhibitor-1 (PAI-1), fibronectin (FN), collagen type I (Col-I), NAPDH oxidase gp91^phox^ (Nox2), Nox2′s cofactor, p47^phox^, Nox4, phosphorylated-nuclear factor kappa-B p65 subunit (NF-κB-p65), and ß-actin were determined on the blots. The antibody information and analysis of the immunostaining bands were described previously [43,44,45].

### 4.11. RNA Preparation and Real-Time RT-PCR Assay

Total RNA from rBMSC cells, either on the sheets or in cell suspension, was extracted using TRIzol reagent (Thermo Fisher Scientific) and PureLink RNA Mini Kt (Life Technologies) according to the manufacturer’s protocols. cDNA was prepared from 1 µg of total RNA using high capacity cDNA reverse transcription kits (Life Technologies). Real time PCR analysis for rat ß-actin (Rn0066869_m1), vascular endothelial growth factor (VEGF, Rn01511602_m1), fibroblast growth factor 2 (FGF2, Rn00570809_m1), insulin-like growth factor-1 (IGF-1, Rn00710306_m1), and hepatocyte growth factor (HGF, Rn00566673_m1) was performed with the TagMan Universal PCR Master Mix using an Applied Biosystems Step One instrument (Applied Biosystems^TM^, Foster City, CA, USA). Samples were run as triplicates in separate tubes to permit quantification of the target gene normalized to ß-actin. Sequences of primers used for this assay were manufactured by Applied Biosystems^TM^.

Total RNA from kidney cortex tissue of each rat was isolated using TRIzol Reagent (Thermo Fisher Scientific). Real-time RT-PCR was performed using the superscript III first-strand synthesis kit, the power SYBR green PCR master mix (Thermo Fisher Scientific), and the ABI 7900 Sequence Detection System (Applied Biosystems), as described previously [46]. Samples were run as triplicates in separate tubes to permit quantification of the target molecules normalized to ß-actin, and analyzed by 2^(−∆∆Ct)^. The relative mRNA levels of the target molecules were expressed relative to the normal control kidney, which was set at unity. Sequences of primers used for rat TGFß1, PAI-1, FN, Col I, and ß-actin were described previously [45,47]. Sequences of new primers, related to regenerative genes such as VEGF, FGF2, HGF, IGF-1, and Pax2, cellular oxidative stress and inflammation such as vanin-1, IL-10, and cellular apoptosis such as TSP-1, Bcl-2, and HO-1 are included in the Supplementary Table S1.

### 4.12. Statistical Analysis 

Data are expressed as mean ± s.d. with n representing the number of animals. Groups were analyzed by one-way ANOVA and subsequent Student–Newman–Keuls or Dunnett’s test for multiple comparisons. *p* < 0.05 was considered statistically significant. The disease-induced increase in a variable was defined as the mean value for the disease control group minus the mean value of the normal control group (100%). The percent reduction in disease severity in a treated group was calculated as follows: (1 − (treated group mean − normal control group mean)/(disease control group mean − normal control group mean)) × 100. The urinary albumin excretion (UAE) values were log_10_-transformed because of skewed distribution. As for a comparison in UAE, the log UAE was considered.

## Figures and Tables

**Table 1:** Functional parameters of rats with chronic glomerulonephritis at week 4.

	NC (*n* = 7)	DC (*n* = 7)	DC + CS (*n* = 7)
B.W. (g)	303.2 ± 27.3	313.2 ± 17.8	310.7 ± 21.1
MAP (mmHg)	91.98 ± 6.97	93.71 ± 10.02	86.85 ± 15.29
Serum BUN (mg/dL)	22.99 ± 1.44	34.68 ± 4.33 *	29.49 ± 2.92 *^#^
Serum Cr (mg/dL)	0.318 ± 0.018	0.386 ± 0.048 *	0.340 ± 0.026 *^#^
K.W./B.W. (mg/g)	6.7 ± 0.3	7.1 ± 0.3 *	6.8 ± 0.5

B.W., body weight. MAP, mean artery pressure. K.W., kidney weight. NC, normal control rats. DC, diseased control rats received sham surgery. DC + CS, diseased rats received rBMSC sheets for 4 weeks. * vs. NC, *p* < 0.05; ^#^ vs. DC, *p* < 0.05.

## Data Availability

Please contact author for data requests.

## Supplementary Materials

The following supporting information can be downloaded at: https://www.mdpi.com/article/10.3390/ijms24043711/s1.
