# Supplementary material for: Engineered Bone Marrow Stem Cell-Sheets Alleviate Renal Damage in a Rat Chronic Glomerulonephritis Model
Source: Int J Mol Sci. 2023 Feb 13;24(4):3711. doi: 10.3390/ijms24043711 (PMC9959772; doi:10.3390/ijms24043711)
Supplement: Supplementary file 1 [file ijms-24-03711-s001.zip › ijms-2156408-supplementary.pdf]

Engineered Bone Marrow Stem Cell-Sheets Alleviate Renal Damage in a Rat Chronic Glomerulonephritis Model

Bin Wang [1,†], Kyungsook Kim [2,†], Mi Tian [1], Sumako Kameishi [2], Lili Zhuang [1],

Teruo Okano [2,3,*] and Yufeng Huang [1,*]

1 Department of Internal Medicine, Division of Nephrology & Hypertension, University of Utah Health Science, Salt Lake City, UT 84132, USA
2 Cell Sheet Tissue Engineering Center (CSTEC), Department of Pharmaceutics and Pharmaceutical Chemistry, University of Utah Health Science, Salt Lake City, UT 84112, USA
3 Institute of Advanced Biomedical Engineering and Science, Tokyo Women's Medical University,Tokyo 162-8666, Japan
* Correspondence: teruo.okano@utah.edu (T.O.); yufeng.huang@hsc.utah.edu (Y.H.); Tel.: +801-585-0581 (Y.H.); Fax: +801-213-2563 (Y.H.)
† These authors contributed equally to this work.

Supplemental Table S1.

Primers used for real time RT-PCR

Gene	Primer	Sequence 5'- 3'
Rat IL-10	Forward<br>Reverse	AAAGCAAGGCAGTGGAGCAG<br>TCAAACTCATTCATGGCCTTGT
Rat Vanin-1	Forward<br>Reverse	GGGAGTTTCAGGTGTTGAG<br>TGAGTGTGCTATGAGGTCTG
Rat Bcl-2	Forward<br>Reverse	ACTCTTCAGGGATGGGGTGA<br>TGACATCTCCCTGTTGACGC
Rat HO-1	Forward<br>Reverse	ATCCCTTACACACCAGCCAC<br>TCCAGAGTGTTCATGCGAGC
Rat TSP-1	Forward<br>Reverse	TCGGGGCAGGAAGACTATGA<br>ACTGGGCAGGGTTGTAATGG
Rat Pax-2	Forward<br>Reverse	CAAAGTTCAGCAGCCTTTCC<br>GTTAGAGGCGCTGGAAACAG
Rat VEGF	Forward<br>Reverse	AACGAAAGCGCAAGAAATCC<br>GCTCACAGTGAACGCTCCAG
Rat FGF2	Forward<br>Reverse	GAACCGGTACCTGGCTATGA<br>CCGTTTTGGATCCGAGTTTA
Rat IGF-1	Forward<br>Reverse	TCAGCGGAGCACAGTACATC<br>GCTTTTACTTCAACAAGCCCACA
Rat HGF	Forward<br>Reverse	GAATGCATGACCTGCAACGG<br>TGTCGGGATATCTTTCCGGC

Supplemental Table S2.

Functional parameters and glomerulosclerosis in diseased rats treated with MCS suspension

	NC (n =7)	DC (N=7)	DC+CS (N=7)	DC+SC (N=7)
B.W.(g)	303.2±27.3	313.2±17.8	310.7±21.1	98.6±11.9
MAP (mmHg)	91.98±6.97	93.71±10.02	86.85±15.29	93.58±14.49
Serum BUN (mg/dL)	22.99±1.44	34.68±4.33*	29.49±2.92*#	33.81±7.86*
K.W./B.W. (mg/g)	6.7±0.3	7.1±0.3*	6.8±0.5	7.6±0.5*#
logUAE (µg/24hr)	1.95±0.09	3.12±0.15*	2.34±0.14*#	3.21±0.13*
Glomerular ECM score (%)	21.1±0.31	38.33±5.13*	25.51±2.76#	37.96±2.63*

NC, normal control rats. DC, diseased control rats received sham surgery. DC+CS, diseased rats received rBMSC sheets for 4 weeks. DC+SC, diseased rats received one-time artery injection of signal cell suspension of rBMSCs for 4 weeks * vs. NC, P<0.05; # vs. DC, P<0.05. B.W., body weight. MAP, mean artery pressure. K.W., kidney weight. logUAE, log10-transfomred urinary albumin excretion (UAE, µg/24h) values. Glomerular extracellular matrix (ECM) score expressed as the percentage of glomerular ECM stained in pink color over the total tuft of glomerulus was described in the method. Those results revealed that treatment with classic MSC suspension via arterially delivery to the kidneys for 4 weeks had no effect on proteinuria and glomerulosclerosis in this model.

Supplemental Table S3.

mRNA expression levels of markers of renal fibrosis, podocyte and tubular injury in diseased rats treated with MSC suspension

	NC (n =7)	DC (N=7)	DC+CS (N=7)	DC+SC (N=7)
TGFß1	1.00±0.10	2.23±0.41*	1.20±0.20#	4.43±0.16*#
PAI-1	1.00±0.11	3.59±0.43*	2.53±0.28*#	8.61±0.40*#
FN	1.00±0.11	1.96±0.14*	0.86±0.14#	3.59±1.21*#
Col I	1.00±0.04	1.86±0.08*	1.02±0.07#	3.87±0.11*#
WT-1	1.00±0.02	0.47±0.06*	0.88±0.01*#	0.54±0.01*
Nephrin	1.00±0.10	0.55±0.10*	1.31±0.12*#	0.34±0.02*#
Podocin	1.00±0.06	0.77±0.01*	1.20±0.14*#	0.73±0.02*
Kim-1	0.99±0.07	3.33±0.04*	1.95±0.07*#	7.22±0.30*#
NGAL	1.00±0.08	2.55±0.02*	1.16±0.02*#	3.20±0.04*#

NC, normal control rats. DC, diseased control rats received sham surgery. DC+CS, diseased rats received rBMSC sheets for 4 weeks. DC+SC, diseased rats received one-time artery injection of signal cell suspension of rBMSCs for 4 weeks. * vs. NC, P<0.05; # vs. DC, P<0.05. The mRNA expression levels of those fibrotic and podocyte and tubular injury markers were determined by real time RT-PCR. Total RNA isolation and real time RT-PCR were carried out as described in the method. These results revealed that treatment with classic MSC suspension via arterially delivery to the kidneys for 4 weeks had no effect on disease-induced elevated mRNA expression of fibrotic molecules and Kim-1 & NGAL, and disease-induced reduction of mRNA expression of WT-1, nephrin and podocin seen in diseased kidneys. Moreover, most of those molecule mRNA expression was even worse than those in untreated diseased rats, further indicating that treatment with classic MSC suspension for 4 weeks had no therapeutic effect, at least, for this model.
